# H.O.P.E. grows: An academic‐public health partnership to reimagine public health services and increase mental health access among socially vulnerable populations

**DOI:** 10.1111/1475-6773.14253

**Published:** 2023-11-20

**Authors:** Victoria C. Scott, Annalise J. Tolley, Jennifer Langhinrichsen‐Rohling, Kayla Walker, Tamikia Greene

**Affiliations:** ^1^ University of North Carolina at Charlotte Charlotte North Carolina USA; ^2^ Mecklenburg County Health Department Case Management & Health Partnerships Charlotte North Carolina USA

**Keywords:** academic‐public health partnership, aligning systems for health, case study, cross‐sector partnership, health equity, integrated care

## Abstract

**Objective:**

To illustrate the process of developing and sustaining an academic‐public health partnership for behavioral health integration through an expansion of the Aligning Systems for Health (ASfH) framework.

**Study Setting:**

Practice‐informed primary data (2017–2023) from the Holistic Opportunity Program for Everyone (HOPE) Initiative based in Charlotte, NC.

**Study Design:**

The unit of analysis in this descriptive case study is inter‐organizational, specifically focusing on an academic‐public health relationship. We illustrate the partnership process across the ASfH four core areas, including key challenges and insights.

**Data Collection:**

Utilized a Critical Moments Reflection methodology and review of HOPE program data.

**Principal Findings:**

(1) Formal partnership structures and processes are essential to monitoring the four ASfH core components for on‐going system alignment. (2) Aligning systems for health principally involves two ecologies: (i) the health program and (ii) the partnership. The vitality and sustainability of both ecologies require continuous attention and resource investment. (3) Relationships rest at the heart of aligning systems. (4) With comparative advantages in research methods, the academic sector is especially poised to collaborate with healthcare systems and human service organizations to study, develop, implement, and scale evidence‐based health interventions.

**Conclusions:**

The academic sector shares overlapping purposes with the public health, healthcare, and social services sectors while providing complementary value. It is a critical sectoral partner in advancing population health and health equity.

**What is known on this topic**

- Cross‐sector partnerships are critical to promoting community health and health equity. However, the pathways to effective and sustainable cross‐sector partnerships are less well understood.
- Aligning Systems for Health is an emergent, prominent theoretical framework for building cross‐sector alignment that requires field‐testing and refinement.
- While the benefits of academic‐public health partnerships are well‐recognized, descriptive studies about how to effectively establish and sustain this type of cross‐sector partnership remain limited.

**What this study adds**

- This study expands the Aligning Systems for Health theoretical framework to include the academic sector and demonstrates the opportunities and value associated with integrating this sector.
- The article illuminates the process and pitfalls of academic‐public health partnership formation, growth, and maintenance; and lends generalizable practice insights for cross‐sector partnerships more broadly.

## 1 INTRODUCTION

Cross‐sector partnerships are essential to improving health equity and population health outcomes.^1^, ^2^ Defined as a formal alliance between two or more organizations representing different sectors of society,^3^ cross‐sector partnerships amplify the potential for social transformation by enabling organizations to pool resources and expertise to address larger social agendas and longer‐term challenges.^4^, ^5^, ^6^ This is particularly true when improving population health and well‐being involves intervening in social, economic, and structural factors that span ecological levels.^2^, ^7^, ^8^, ^9^, ^10^

Two sectors with a long‐standing history of collaboration to advance population health are academia and public health departments (i.e., local and state government health agencies). Academic‐public health partnerships were spotlighted at the dawn of the twenty‐first century when the Institute of Medicine underscored the importance of this particular collaboration.^11^ Key advantages of these partnerships to public health departments include buttressed workforce development, greater access to subject matter experts, and increased use of evidence‐based practices; collectively, these contribute to enhanced health service capability and patient care quality.^12^, ^13^, ^14^ For the academic sector, these partnerships engender opportunities for research, external funding, and student field‐based training.^15^, ^16^, ^17^ They also afford academics a pathway to mission‐driven, applied contributions. At the community level, the distal impacts of academic‐public health partnerships include greater community engagement, improved population health, and increased health equity.^18^, ^19^, ^20^

While academic‐public health partnerships are full of potential, the processes of establishing and sustaining these partnerships can be complex, daunting, and elusive.^21^, ^22^ The lack of applied research demonstrating best practices required to grow and sustain cross‐sector partnerships between academia and public health is more than a mere literature gap. Poorly supported and maintained partnerships may result in unintended consequences that place an unfair burden on one partner, tarnish trust among stakeholders, and compromise implementation fidelity.^23^ Consequently, this may impede progress toward addressing health inequities. To prevent these unintended consequences, identifying factors underlying successful academic‐public health partnerships is imperative. To this end, this article provides a descriptive study of an academic‐public health partnership designed to promote behavioral health integration. We draw upon the Aligning Systems for Health (ASfH) framework^24^ to illustrate the process of partnership formation, growth, and continuity across a multi‐year journey.

### 1.1 ASfH framework

ASfH is an emergent cross‐sector collaboration framework for sustainable progress toward advancing population health and community well‐being. It was developed by Robert Wood Johnson Foundation (RWJF) and is based on three decades of consolidated learnings pointing to the need for more aligned and enduring cross‐sector partnerships to improve national health outcomes.^22^, ^24^ Four alignment areas comprise the heart of the framework: shared purpose, governance, finance, and data and measurement. The framework initially identified three sectors as central partners (public health, healthcare, and social services) and is actively being tested and refined.^25^

In this article, we expand the ASfH framework to include academia as a fourth key sector. We describe a longitudinal process of aligning the four core components in the context of an academic‐public health partnership focused on increasing mental health equity through a health initiative called the Holistic Opportunity Program for Everyone (HOPE). We provide candid descriptions of partnership and program implementation processes, with the aim to (i) illuminate stepping stones for other system partners embarking upon similar work, and (ii) illustrate the value‐add of the academic sector as a key partner to improve community health and health equity.

## 2 FOUR CORE COMPONENTS TO SYSTEMS ALIGNMENT: BUILDING THE HOPE PARTNERSHIP

The following section describes how we aligned our academic‐public health partnership across the four ASfH areas. A summary of key insights for each ASfH area is available in Table 1. General partnership benefits with illustrative HOPE‐specific examples are depicted in Table 2. Before detailing the development of each ASfH area, we provide a description of HOPE—the case unit for this descriptive study.

**TABLE 1:** Insights for cross‐sector alignment across the four ASfH components and example practical steps for aligning.

Core components	Insights for aligning	Example action steps
Shared purpose	1: Establish a shared purpose early on to serve as a compass for the partnership 2: Patiently involve key stakeholders in iterative discussions to develop a clearly articulated shared mission and logic model 3: Return to the program logic model to frame discussions and decisions in meetings. As needs evolve, routinely revisit the program logic model and mission statement for alignment	Establish the shared mission/vision for the partnership Develop a program logic model to operationalize the mission/vision Create branding materials to reflect the partnership's shared purpose (e.g., name, logo, website, posters, swag) Revisit and update the shared purpose routinely Build organizational buy‐in so that the shared purpose outlasts any changes in personnel
Shared governance	1: Use an integrative partnership approach rooted in empowerment to foster a culture of inclusion and distributive leadership 2: Program governance interacts with organizational governance. Attend to the flow of information laterally (communication among partners within the program) and vertically (communication between the program and organizations) 3: Provide organizational decision makers with on‐going relevant information, particularly about program successes, is imperative for program sustainability	Identify backbone organization to support cross‐sector partnership Specify infrastructure for collaboration (roles and responsibilities, file sharing system, meeting cadence), including key communication pathways Build mechanisms to ensure team member accountability Integrate community voice into governance infrastructure
Shared finance	1: It is more difficult to obtain funding for partnership than for program activities; yet, the partnership is essential to improve the implementation and effectiveness of the program 2: Prioritize internal funding (over external funding) to institutionalize support for partnership activities 3: Shared financing is imperative to joint commitment. Significantly imbalanced institutional financing may lead to disproportional investment. 4. Finances are often tied to program deliverables or milestones. These need to be aligned with program development as well as leadership priorities	Discuss and explore financing options (e.g., internal institutional funding, external funding) Establish a process for monitoring partnership finances Align partnership activities with financial cycle/timeline Advocate for funding opportunities to be created that support the partnership in addition to the program/desired outcomes
Shared data & measurement	1: The location of where program data are housed can have important practical implications for program and performance monitoring if the data are not readily accessible to all cross‐sector partners 2: When data are housed in different sectors, it is critical to establish infrastructure whereby data are accessible across partners. This infrastructure may be formal (e.g., data sharing agreement, intranet access), or informal (e.g., routine aggregate data updates) 3: Despite a shared purpose, organizational partners may each have unique goals for data use. Ensuring appropriate access to the data enables the partnership to realize the upper potential of program data	Establish performance metrics Identify or develop a data management system Develop data dashboard Determine cadence and process for reviewing program data, including data quality assurance Develop a data sharing agreement

**TABLE 2:** General benefits of academic‐public health partnerships and illustrative examples through HOPE.

General benefits	Examples from HOPE
**Benefits to the public health department**	
Access to peer‐reviewed research to guide practice and increase the use of evidence‐based decision‐making	The academic team reviewed existing research literature on clinical uses of the PHQ‐2 to determine the best referral score for the PHQ‐2 in the HOPE population
Access to subject matter experts	Faculty with expertise in implementation science, quality improvement, community engagement, mental health screening, and integrated care delivery provided ongoing consultation. University faculty and students led assessments of organizational readiness, a central HOPE data collaboration
Resources for public health workforce development	Using results from the readiness assessment, workforce development activities were tailored to address underdeveloped staff competencies through staff‐requested trainings (e.g., Motivational Interviewing). Organizational readiness for HOPE among public health staff has steadily risen across the 3 years of the program (mean score: 4.85, 5.20, 5.72 on 7‐point scale)
Additional funding sources	The academic team has provided university seed funding, included HOPE in broader grant development, and accessed graduate assistantship funding to support program implementation and expansion
Enhanced credibility via professional conference presentations and publications	Insights about HOPE have been disseminated at local, regional, and national multidisciplinary conferences. In 2022, the HOPE Leadership Team delivered a four‐part symposium at the American Public Health Association Conference. This represented the first national professional conference presentation for all public health co‐authors
Increase organizational capacity via student interns	Over 20 student interns have collaborated on HOPE and have spearheaded the creation of HOPE marketing materials (i.e., HOPE Bulletin), provided essential administrative support, and expanded the evaluation capacities of the public health department
**Benefits to academia**	
Hands‐on opportunities for student development via student field placements	Undergraduate, Master's, and Doctoral students in public health, organizational sciences, clinical psychology, community psychology and sociology have participated on the HOPE team. As a result of their involvement, students have received summer internships, expanded opportunities for research, and extended community engagement experience
Access to public health data for applied research related to health disparities and inequities	Theses and dissertation projects have utilized HOPE readiness data as well as program outcome data; additional projects are in development
Community‐engaged and mission‐driven scholarship	Through an integrative partnership approach with MCPH, faculty and students are advancing the HOPE mission and contributing to community health improvement
External funding	HOPE has provided multi‐year stipends for two faculty and one graduate assistant
Scholarly products	Numerous professional conference presentations, scholarly reports, and manuscripts have been realized through the HOPE partnership
**Benefits to the community**	
Improved healthcare access and care quality	MCPH clients have greater access to evidence‐based, culturally responsive behavioral health services. Between 2021 and 2022: 19,154 clients received mental health screening, 417 clients received education or linkages to community resources, and 44 clients in crisis received immediate intervention
Improved population health and health equity	MCPH hired two full‐time behavioral health providers and integrated behavioral health services into multiple public health clinics. This is a pioneering step toward improved population health and health equity
Increased community engagement	Currently in progress, HOPE aims to include the patient voice in leadership meetings to shed light on root causes of barriers to well‐being and to provide insights for service delivery improvement

### 2.1 Case unit: the Holistic Opportunity Program for Everyone (HOPE)

In recognition of the importance of academic‐public health partnerships to advance population health, particularly in underserved communities, the Academy for Public Health Innovation (APHI) was formed in Mecklenburg County in 2016. APHI is a formal partnership between the Mecklenburg County Public Health (MCPH) department and the University of North Carolina at Charlotte (UNCC). Through APHI, the authors of this paper convened with a shared vision to integrate behavioral health services into public health clinics as a critical step toward holistic wellness for underserved residents in Mecklenburg County.^26^ This shared vision led to the development of HOPE.

HOPE includes three core service components: (i) universal administration of a depression symptom screening measure, (ii) referral to on‐site behavioral health providers, and (iii) referral to community resources. HOPE services are currently integrated into five clinics: two Family Planning Clinics, one HIV/STI clinic, and two Special Supplemental Nutrition Programs for Women, Infant, and Children (WIC) clinics. The following sections detail our journey to align the four ASfH core components as we established, embedded, and expanded HOPE across these disparate clinics.

### 2.2 Shared purpose

Shared purpose refers to the ways in which partners define goals, vision, mission, and outcomes.^25^ A shared purpose is essential for the long‐term success of any cross‐sector partnership.^24^, ^27^, ^28^

#### 2.2.1 Shared purpose in action

The HOPE Leadership Team is composed of individuals representing the public health department (MCPH) and academia (UNCC). The public health team consists of executive (TG), clinic, behavioral health, and informatics leadership. The academic team is composed of two faculty with community, clinical, and implementation science expertise (VS & JLR), a graduate research assistant (AT), and an undergraduate honors student (KW). At the outset of our academic‐public health partnership, our Leadership Team focused on developing a HOPE program mission statement. The process involved multiple joint discussions facilitated by an executive leadership member (TG) from the public health department. The mission statement succinctly concretized the program's purpose. Additionally, it contributed to shared program ownership, which was particularly important given the newly formed HOPE Leadership Team. Next, we aligned the name of the initiative (HOPE) with its mission. The naming and subsequent program branding helped to establish and disseminate the HOPE mission. Institutionalizing the shared purpose of HOPE also ensured that the vision was not bound to any specific public health or academic personnel. This strategy is especially salient for cross‐sector partnerships with higher staff turnover. For example, in the case of academic‐public health partnerships, students can be expected to graduate or transition from projects. Public health personnel can also shift positions or change focus per emergent health department priorities.

Developing a shared logic model was another critical step toward formalizing the explicating our shared purpose. The process of articulating the HOPE logic model necessitated collaborative discussion, which shed light on implicit assumptions held by team members. For example, we unearthed significant variability in initial expectations about plausible program effects held by various HOPE Leadership Team members. Some members held modest expectations (e.g., our health department will be better at identifying patients with depression) while others held ambitious hopes (e.g., the prevalence of depression will decrease in Mecklenburg County). Solidifying the program logic model required agreement about the program's theory of change, encouraged the development of realistic short and long‐term program outcomes, and provided the team with greater insight into program limitations and opportunities. Further, establishing the mission, shared language, and logic model early in the partnership helped us weather the COVID‐19 pandemic and sustain the initiative through clinic staff changes.

#### 2.2.2 Key challenges

Health and human service programs often emerge from the rough and tumble of political support, opposition, and bargaining for resources and attention. As a result, health promotion programs must balance program aims with the diverse and evolving interests of multiple stakeholders. This has been a significant challenge across the initiative's lifespan. For example, while HOPE was originally created with the purpose of supporting perinatal women exclusively, some Leadership Team members advocated for expanding screening to include all individuals receiving any type of clinic services. To determine if there was enough stakeholder support to enlarge our shared purpose, the team conferred with staff and considered benefits and concerns. Ultimately, we decided to expand the target population. Corresponding changes to the mission statement, logic model, and updates to anticipated program milestones and deliverables were then needed to reflect this decision and to maintain program coherence across time.

Additionally, while shaping our shared purpose, we experienced the tension between “planning” and “doing,” with many team members feeling the pressure to get things done through quick program implementation. This challenge was addressed through explicitly acknowledging the tension. We also quantified some of our planning activities as “products” to meet annual program reporting requirements (e.g., completed mission statement and program logic model). Further, we decided to increase the cadence of partnership meetings to routinely address both planning and implementation activities.

### 2.3 Shared governance

Governance structures range from informal agreements among a few parties to formal structures established across large organizations.^25^ Shared governance is widely recognized as a key determinant of partnership effectiveness and includes infrastructure, leadership, defined roles, and processes for decision‐making.^22^, ^25^

#### 2.3.1 Governance in action

**FIGURE 1:**
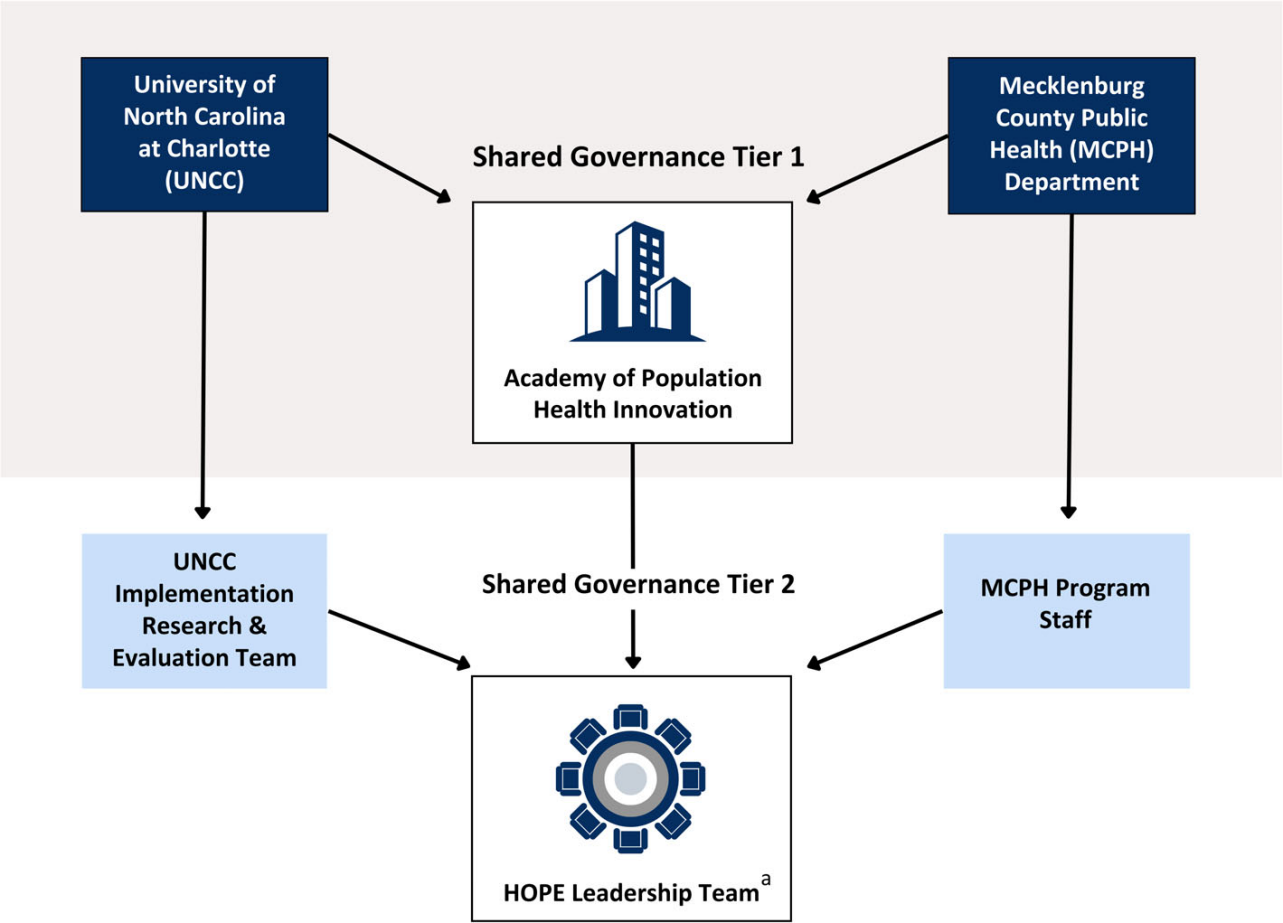
HOPE governance structure. While the delineation of the HOPE governance structure presents implementation research and evaluation on one side and the HOPE program on the other, these areas of practice are inextricably linked and are foci of concern shared by all members of the interdisciplinary HOPE Leadership Team. ^a^The circular icon accompanying the HOPE Leadership Team is presented in greater detail in Figure 2.

HOPE involves two tiers of governance that operate in tandem: a formal governance structure between the university and the public health department and a semi‐formal governance structure at the program level known as the HOPE Leadership Team (Figure 1).

##### Governance Tier One

It is common for cross‐sector partnerships to have a backbone organization that can leverage existing resources, capacities, and infrastructure while maintaining a degree of separation,^25^ which serves to impartially propel the initiative forward. For HOPE, that organization is APHI, which provides formal governance for the partnership. The APHI Executive Team is represented by leadership from both the university (i.e., Professor of Public Health Sciences, Public Health Sciences Department Chair, APHI Operational Director) and the public health department (Director, Deputy Director, Director of Administrative Services and Compliance, Medical Director).

Operationally, APHI involves routine meetings between institutional executive leadership, joint annual strategic planning, an operational budget, and defined roles and responsibilities. The APHI backbone structure encourages cross‐sector (horizontal) information exchange at the executive level, which in turn, determines priorities and resource allocation within institutions (and thus within nested programs such as HOPE). Advantageously, an APHI administrative officer (VS) also serves as the university lead for HOPE. This organizational arrangement enhances vertical information exchange (i.e., between HOPE leadership and APHI executive leadership).

##### Governance Tier Two

At the HOPE program level, the governance structure is semi‐formal and is led by the Assistant Health Director (TG). HOPE Leadership Team members have defined roles and responsibilities shaped by program and partnership needs. While participation in HOPE is not mandated for team members, it is perceived favorably by key decision‐makers in Tier One of governance (to whom all team members are ultimately accountable). For example, public health staff include their involvement with HOPE on annual performance evaluations to demonstrate how they go above and beyond in their roles. Similarly, university faculty can showcase their involvement with HOPE to illustrate their contribution to the university's community engagement mission and to strengthen support for faculty promotions and salary increases.

The scope of university services provided to the health department is specified in a project contract drafted by Tier Two leadership and then negotiated and executed annually by Tier One leadership. Services include training, implementation consultation and support, and program evaluation. As an example, for the past 4 years, the partnership contract has included an assessment of organizational readiness for HOPE among public health clinics. This assessment activity continues to be delineated in contract negotiation because it enables annual, practice‐based changes that are data‐informed.

**FIGURE 2:**
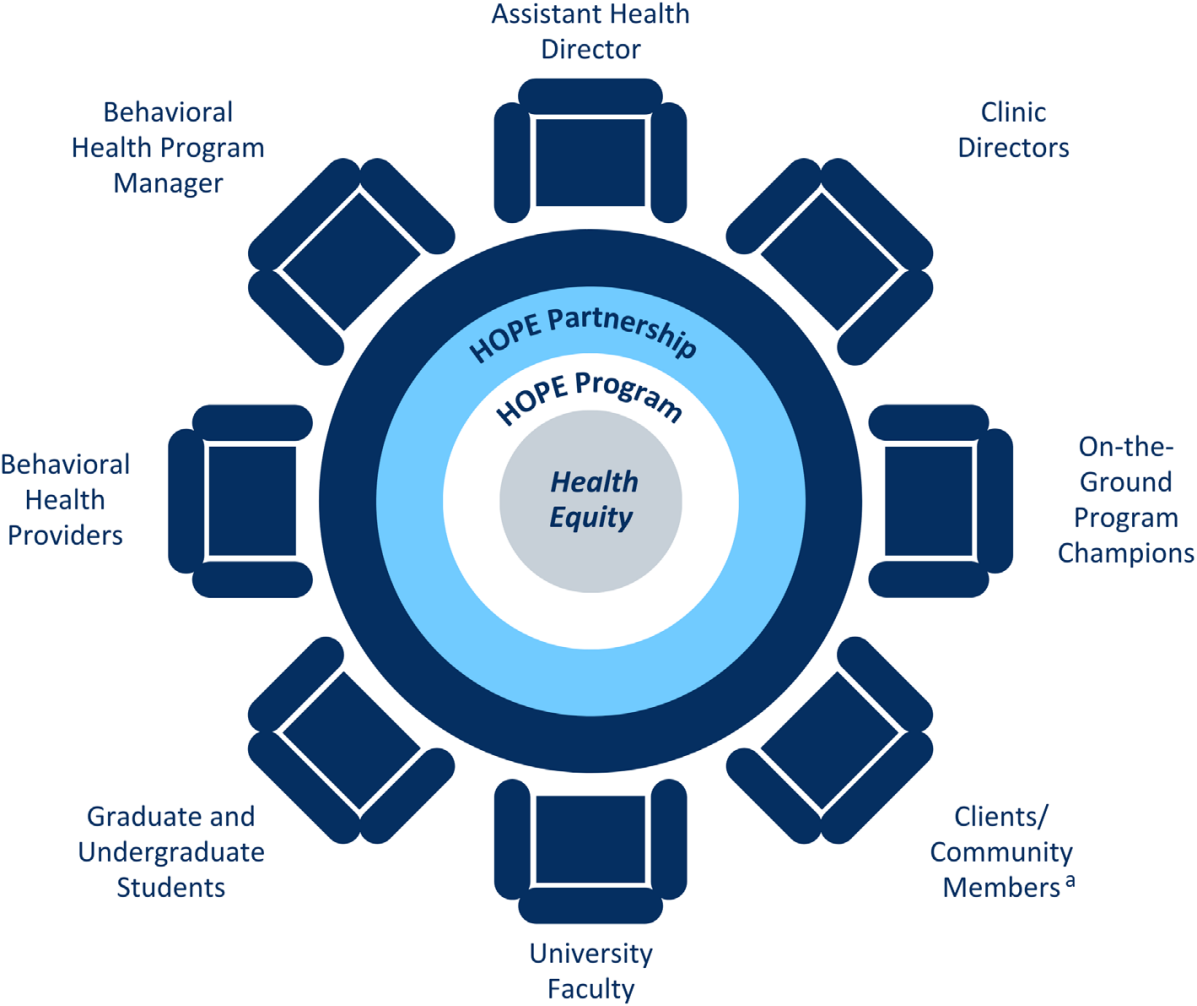
This figure depicts the metaphoric table we use to illustrate our integrative academic‐public health partnership approach. Regardless of formal titles, all members have a seat at the table. Decision‐making and accountabilities are shared. The HOPE Leadership Team is composed of individuals representing the public health department (MCPH) and academia (UNCC). Specifically, the public health team involves executive (TG), clinic leadership, behavioral health and informatics leadership. The core academic team involves two faculty (VS & JLR), a graduate research assistant (AT), and an undergraduate honors student (KW). ^a^Clients/community members are not yet active participants of the Leadership Team.

Traditionally, academic‐public health collaborations have been akin to a bridge between two disparate domains of “research” and “practice.” In this model, cross‐sector engagement is often characterized as functionally transactional (e.g., one partner provides data, the other partner analyzes the data and publishes findings). A critical governance feature and strength of HOPE is our integrative (rather than transactional) collaboration approach. Rooted in the principle of empowerment, we welcome the expertise and perspectives of all team members. We view the partnership as a metaphoric table at which all members have a seat and share decision‐making and accountabilities (Figure 2).

Across time, we have found that our integrative partnership approach fosters distributed leadership, promotes a culture of inclusiveness, and yields creative solutions. For example, since HOPE is implemented in WIC clinics, data are stored with the state and are largely inaccessible in real‐time. In response, HOPE informatics leadership drew on research literature provided by university partners and consulted public health key performance measures to develop a web‐based platform that enabled key data to be filtered into a real‐time data dashboard. Without the interdisciplinary team, this solution would not have emerged.

#### 2.3.2 Key challenges

Institutional priorities and issues both across partnership sectors and within sectors can facilitate or impede implementation success. For example, the sustainability of HOPE is tied to funding support provided by APHI (backbone structure) and can be influenced by other existing UNCC‐MCPH partnerships and programs. A soured UNCC‐MCPH partnership occurring in a separate program can have a reverberating effect on HOPE. Additionally, there are often competing priorities in MCPH, such as public health community crises (i.e., COVID‐19 and MPox outbreaks) or internal organizational changes that can limit staff's capacity to dedicate energy toward implementing and expanding the initiative.

### 2.4 Shared finance

Finance is defined as the acquisition and management of funds to support effective, sustainable collaborations through incentives and accountabilities.^25^ For HOPE to grow, we require financing for both the program and the partnership.

#### 2.4.1 Finance in action

Initially, our academic‐public health collaboration was financed by a private foundation grant (RWJF) along with in‐kind university support (pro‐bono faculty and student‐in‐training time). The RWJF grant was obtained by a public health clinic director, with grant writing support provided by university faculty. This initial 1‐year RWJF funding enabled us to conduct a pilot study, thus launching the program‐level academic‐public health partnership. After the successful pilot project, a shift was made to funding provided by the public health department (county funding), with continued in‐kind support from the university (i.e., students‐in‐training, faculty time). Operationally, the public health department funds key university collaborators via the backbone organization (APHI) and public health team members directly via employee payroll. The shift from external grant funding (RWJF) to institutional, internal funding has pivotally supported the steady development of our partnership by providing sustained resources.

#### 2.4.2 Key challenges

Internal financing has benefits to cross‐sector partnership; however, the perennial nature of the HOPE budgeting process is an on‐going challenge. Each year, HOPE Leadership from both institutions (TG, VS, JLR) are required to create a budget for continued services provided by the university to the public health department, which is subsequently negotiated by APHI executive leadership. We have found it valuable to hold joint meetings involving leadership from Tier One and Tier Two governance to align expectations and to subsequently incorporate executive leadership language and priorities into budget proposal.

An emerging financing challenge is how to support program expansion. New program needs include hiring additional behavioral health providers, conducting on‐going staff trainings, and incorporating patient voice. We have explored a combination of internal and external funding sources but have currently prioritized securing internal funding for the reasons outlined above.

### 2.5 Shared data and measurement

Program data are fundamental to program monitoring and improvement and, ultimately, to discerning program effectiveness.^29^, ^30^, ^31^ Shared data and measurement involve close collaboration among partners to identify indicators and to develop a measurement system to coordinate activities and track joint progress effectively.^25^ Who, what, and how data are collected, shared, and utilized can also shed light on the strength and health of the partnership.

#### 2.5.1 Data and measurement in action

An early priority for HOPE was to develop an infrastructure for data collection and monitoring. Two highly engaged MCPH informatics staff were recruited to the HOPE Leadership Team. In a collaborative and iterative fashion, key program metrics were identified, incorporated into the public health electronic health record (EHR) system, and reflected in a data dashboard. While the dashboard was jointly designed, it is housed in the public health data system owned by the public health department. As a result, the university team relies on public health staff to send program data updates bi‐weekly. Meanwhile, the university team has ownership over separate HOPE data, which reflect organizational readiness for HOPE and are used for program monitoring and improvement. It is incumbent upon the university team to share the readiness assessment data with public health staff in a digestible and actionable way to drive data‐informed practice change. We believe shared ownership of both the program and the quality improvement data between sectors valuably operationalizes shared trust and accountability. Developing strategies to share data and discuss it together has thus become a key indicator of a healthy HOPE partnership.

#### 2.5.2 Key challenges

While data sharing has contributed to the success of our cross‐sector partnership, it has not been without difficulties. Having different data housed and managed by different sectors necessitates greater communication, coordination, and follow‐through. To date, HOPE data sharing has required manual effort. Specifically, a public health team member summarizes trends or sends a screenshot of the data dashboard to the university partners, as there are barriers to outside partners accessing the health department's EHR. Data access permissions are a commonly reported barrier to data sharing.^32^, ^33^, ^34^ Past experience with cross‐sector aligning for health has demonstrated that when both partners are able to rapidly synthesize and view program and partnership data, it has a meaningful impact on community health improvement.^35^ Enhancing our ability to share data bi‐directionally is a growth edge for our partnership.

## 3 DISCUSSION

While cross‐sector partnerships are widely recognized as essential to advancing health equity, the pathways to effective and sustainable cross‐sector partnerships are less well understood. Partnership alignment is shaped by sector‐specific characteristics (structure, culture, resources) and setting context. This article uses practice‐based examples to illustrate issues and solutions that arise when establishing shared purpose, governance, finance, and data and measurement through an academic‐public health partnership. It also underscores the value of including academia as a key cross‐sector partner in ASfH. Our multi‐year effort has resulted in four major insights about cross‐sector partnerships.

### 3.1 Practice insight one: monitor and anticipate fluctuations in ASfH areas

The sustainability of any partnership cannot be assumed. Partnership sustainability is a perpetual consideration for HOPE, particularly as we face concerns regarding continued funding, personnel turnover, competing priorities, and adaption to an ever‐changing context. However, we believe that when partnerships invest in aligning the four ASfH areas, the partnership is better positioned to outlast unforeseen threats to sustainability. Routinizing processes and developing appropriately agile structures within each ASfH component can serve to institutionalize the partnership, thus decreasing its dependence on any particular person, funding stream, or timeline. Still, changes will indubitably impact the partnership and require tailored strategies to ensure ASfH components remain in alignment (as they are dynamic). To identify which tailored strategies are needed for continued alignment, routine assessments of partnership readiness are critical. Readiness assessments, such as those administered by our team, can proactively identify partnership needs and ensure that quality improvement and self‐reflection is embedded within the partnership culture. This will increase the likelihood that a healthy partnership will endure.

The processes of aligning the ASfH areas commonly occur in tandem; however, the relative importance of each component fluctuates across the partnership lifespan. Early in the partnership, we focused our energy and resources on establishing shared purpose and shared governance. This made explicit *why* we were working together, *who* was important to have at the table, and *how* we would work together. Subsequently, we shifted to issues of data sharing and measurement. In this stage, we addressed *what* defines program success and we established a data management infrastructure to support program implementation and monitoring. Similarly, financing has changed over time. While we originally relied on external funding, we shifted to internal funding to ensure the sustainability of the program and the partnership. This shift allowed us to more directly align our activities and outcomes with organizational resources and priorities. We continue to renegotiate our academic‐public health HOPE contract on an annual basis. Given the many pathways to partnership formation, the sequence of aligning the ASfH areas will naturally vary from partnership to partnership.^24^ However, partners can plan proactively by anticipating times when select components will need greater attention and by incorporating formal structures and processes to monitor the four areas.

### 3.2 Practice insight two: effective alignment rests on two ecologies

ASfH principally involves (at least) two ecologies: (i) the health intervention/program (e.g., HOPE), and (ii) the partnership. These two ecologies are appropriately intertwined and thus are easily conflated or conceptualized as a single ecology. In such circumstances, cross‐sector partnerships may be conceptualized as task‐focused, whereby conversations primarily serve the task (i.e., health intervention/program) rather than the partnership. Associated questions may be: *What is the aim of the intervention* (purpose)? *What funding is needed to support and sustain the intervention* (financing)? *Who will do what to support program implementation efforts* (governance)? *What are indicators of program success* (data & measurement)? The tendency for partners to focus on the health intervention is a natural response to the pressure of external accountabilities.

However, an alignment pitfall is to prioritize the health intervention/program over the needs of the partnership. Our journey to HOPE has illuminated a conceptualization of ASfH that is *relationally‐focused*, in which partners give parallel priority to shaping and nourishing the relationship. This paradigm shift opens partners to conversations about additional efforts required to *sustain* an effective partnership. For example, *What is the aim of our partnership* (purpose)? *What funding is needed to support and sustain an effective partnership* (financing)? *Who will do what to support the partnership* (governance)? *What are the indicators of partnership success* (data and measurement)? We posit that both ecologies (program *and* partnership) are critical to ASfH and that the vitality and sustainability of both ecologies requires continuous monitoring and resource investment.

### 3.3 Practice insight three: relationships are at the heart of aligning and sustaining partnerships

Our experience across decades of partnering cross‐sectorally affirms that relationships rest at the heart of aligning systems. Similarly, Miller and colleagues^36^ analyzed 208 health and human service partnerships and found that the most important determinant of successful partnerships was the relationship quality between partners. A decade prior, Kania and Kramer^37^ identified “continuous communication” to foster trust as one of the five essential conditions to successful collective impact initiatives. A noteworthy evolution of the ASfH framework is an explicit recognition of relationships in aligning systems. Specifically, the most recent rendition of the ASfH model highlights the importance of “trust” and “power dynamics,”^38^ whereas these concepts were discussed in the article text but omitted from the original ASfH figure.^24^ There is a growing recognition of the centrality of relationships to ASfH: relationships are the grease that turns the cogs of the four ASfH alignment areas. In our own academic‐public health partnership, the following relational characteristics have made for effective partnering: trust, authenticity, transparency, radical candor, and mutual support. Cultivating these characteristics has required intentionality and investment (e.g., conversations about shared values, leaning into difficult/sensitive conversations, and protecting time for shared meals and informal catch‐ups).

Another critical facilitator to robust relationships is assessing and building readiness for cross‐sector partnership. Partnership readiness is shaped by both ability (capacity) and willingness (motivation) of partners to collaborate. In our own practice, we have utilized a readiness diagnostic scale (e.g., Readiness for Cross‐sector Partnership Questionnaire^39^) to assess the readiness of stakeholders to work together to advance and sustain a health intervention. Given our experience, we assert that other cross‐sector partners can promote partnership effectiveness by systematically assessing partnership readiness and jointly reviewing readiness data to generate strategies for strengthening the partnership.^39^ A recommended precursor to administering a partnership readiness assessment is to establish shared partnership values. These values center what is important to the relationship and can help partners weather difficult conversations and significant intervention‐related challenges.

### 3.4 Practice insight four: academia is a key sector to ASfH

We strongly recommend the integration of the academic sector into the ASfH Framework. The academic sector has a rich history of partnering with healthcare, public health, and social service systems to promote community health. Advancing community health outcomes requires a better understanding of the effectiveness of health interventions, including what works, when, how, and for whom. With comparative advantages in research methodology, the academic sector is especially poised to collaborate with healthcare systems and human service organizations to study, develop, and scale evidence‐based health interventions. Health‐focused research faculty have deep knowledge about an array of health topics. Research faculty also lend expertise in statistics, evidence‐based practice, data management, evaluation, and grantsmanship. Further, the academic sector can support workforce development by mentoring students interested in health, medical, and human services careers. Lastly, through academic‐health system partnerships, the academic sector can enhance health system capacities through no‐cost or low‐cost student placements. Healthcare facilities commonly lack the financial resources needed to engage health providers in evidence‐based practices and quality improvement initiatives.^40^

### 3.5 Limitations and future considerations

This article provides a descriptive account of an academic‐public health partnership. Some limitations are important to note. First, the insights we offer may not generalize to all academic‐public health partnerships nor to other cross‐sector partnerships. Academia and public health are not homogenous sectors. Second, ASfH recognizes the importance of engaging community members as key stakeholders, signaling a growing recognition of the importance of collaboration and community voice in scholarship, training, and practice. However, as HOPE is still working to engage our clients in health service delivery improvement, the salience of the community voice is not illustrated in this article. Lastly, we highlighted key aspects of aligning the ASfH areas, but our coverage is not comprehensive. Partnership development is indeed a process. Additional research is needed to ascertain the most critical alignment activities under each ASfH area and whether a particular sequence of alignment is especially advantageous. Further, more research is needed to understand how different academic‐public health partnership models contribute to the goals of community health improvement. The *Aligning Systems for Health Learning System* ^25(p421)^ provides a useful heuristic for how research and practice can be consolidated to better understand what works, for whom, and under what circumstances.

## 4 CONCLUSION

This article makes three important contributions to the field. First, it offers a rich description of a multi‐year effort to align two systems across the four ASfH core areas. Practice‐based examples critically inform advances in the science and application of ASfH, a prominent but relatively new framework in the field. A longitudinal examination of aligning efforts reveals the ebb and flow of partnerships and how partners can weather significant internal and external factors. Second, this study expands the ASfH framework to include the academic sector and demonstrates both the value and possibilities of integrating this sector. Third, this article illuminates the process and pitfalls of academic‐public health partnership formation, growth, and continuity. While the benefits of academic‐public health partnerships are well‐recognized, descriptive studies about how to effectively establish and sustain these partnerships remain limited.

## FUNDING INFORMATION

The Holistic Opportunity Program for Everyone (HOPE) is funded by the Academy for Population Health Innovation and the Mecklenburg County Public Health Department.
